# Arsenic exposure to mouse visceral leishmaniasis model through their drinking water linked to the disease exacerbation via modulation in host protective immunity: a preclinical study

**DOI:** 10.1038/s41598-023-48642-z

**Published:** 2023-12-05

**Authors:** Ghufran Ahmed, Fauzia Jamal, Ritesh K. Tiwari, Veer Singh, Sachchida Nand Rai, Sanjay K. Chaturvedi, Krishna Pandey, Santosh K. Singh, Ashish Kumar, Shyam Narayan, Emanuel Vamanu

**Affiliations:** 1 Department of Microbiology, Rajendra Memorial Research Institute of Medical Sciences, Patna, 800007 India; 2 Department of Biochemistry, Rajendra Memorial Research Institute of Medical Sciences, Patna, 800007 India; 3 Centre of Experimental Medicine and Surgery, Institute of Medical Sciences, Banaras Hindu University, Varanasi, 221005 India; 4 Department of Clinical Medicine, Rajendra Memorial Research Institute of Medical Sciences, Patna, 800007 India; 5 Faculty of Biotechnology, University of Agricultural Sciences and Veterinary Medicine of Bucharest, 011464 Bucharest, Romania

**Keywords:** Ecology, Environmental sciences, Medical research, Molecular medicine

## Abstract

A large body of evidence has shown a direct link between arsenic exposure and drug resistance to *Leishmania* parasites against antimonial preparations in visceral leishmaniasis (VL) hyper-endemic regions, especially in India and its sub-continent. However, the implicated roles of arsenic on the VL host, pathophysiological changes, and immune function have not yet been clarified, particularly at the reported concentration of arsenic in the VL hyper-endemic area of Bihar, India. Herein, we exposed the mouse VL model to arsenic (0.5 mg/L to 2 mg/L) through their drinking water and analyzed its effect on T cells proliferation, Th1/Th2-mediators, MAPK signaling cascade, and parasite load in preclinical models. Coherently, the parasite count in Giemsa stained spleen imprint has been investigated and found significant positive associations with levels of arsenic exposure. The liver and kidney function tests (AST, ALT, ALP, BUN, Creatinine, Urea, etc.) are apparent to hepatonephric toxicity in arsenic exposed VL mice compared to unexposed. This observation appears to be consistent with the up-regulated expression of immune regulatory Th2 mediators (IL-4, IL-10, TGF-β) and down-regulated expression of Th1 mediators (IL-12, IFN-γ, TNF-α) with a suppressed leishmanicidal function of macrophage (ROS, NO, iNOS). We also established that arsenic exposure modulated the host ERK-1/2 and p38 MAPK signaling cascade, limited T lymphocyte proliferation, and a lower IgG2a/IgG1 ratio to favor the *Leishmania* parasite survival inside the host. This study suggests that the contorted Th1-subtype and exacerbated Th2-subtype immune responses are involved in the increased susceptibility and pathogenesis of *Leishmania* parasite among subjects/individuals regularly exposed to arsenic.

## Introduction

Leishmaniasis comprises an array of clinical manifestations induced by over 21 protozoan parasites of the genus *Leishmania*. The disease is endemic in tropical and subtropical regions of the world and highly prevalent among the economically lower strata of the societies. The Indian sub-continent, especially India, Bangladesh, Nepal, and Bhutan, harbors more than 73% of the total global burden of visceral leishmaniasis (VL), a life-threatening form in over 95% of cases, if not treated^1–3^. About 90% of India's total reported VL arises in the state/province of Bihar and its adjoining regions (Jharkhand, West Bengal, Utter Pradesh), and more than half of the Bihar population (62.5 million) are at the risk of *Leishmania* parasite infection^4–6^. Further, co-infections like VL-HIV, VL-TB, and Post Kala-azar Dermal Leishmaniasis (PKDL) have been alarming over the last two decades in VL endemic regions, especially in Asia and Africa. In this circumstance, chemotherapy is the legitimate option to manage and keep the disease under control. However, the existing chemotherapeutics are small in numbers (Sodium-stibogluconate, Amphotericin-B, Miltefosine, Paromomycin), expensive, in addition to the non-availability of any licensed vaccines for human use^7,8^. Further, the emergence of drug resistance *Leishmania* parasite to these therapeutics significantly reduces its practicality, like the efficacy of Sodium-stibogluconate (SSG) has decreased by more than 64% in the Indian sub-continent and, no longer recommended by clinicians in this region^9,10^. Likewise, the efficacy of Amphotericin-B (AmB) and its liposomal form (AmBisome) and Miltefosine have also been decreasing substantially^11,12^.

Additionally, the emergence of arsenic contaminations in the groundwater of VL endemic regions and its direct involvement in SSG unresponsiveness against *Leishmania donovani*, as we and some other groups demonstrated in experimental settings^10,13^. Arsenic and antimony are similar chemical elements; exposing the *Leishmania* parasite to arsenic within the host could allow the *L donovani* to grow resistant to antimonial preparation^14^. Previously, a pilot scale study was conducted with cohort of SSG/other drug treated VL patients to evaluate the possible role of arsenic in development of PKDL and found significant positive associations^15^. Arsenic has been proven to have a deleterious impact on innate and adaptive immune responses, leading to an increased risk of infections and chronic diseases, including VL, PKDL, and various cancers^16^. It has been reported that arsenic has an immunosuppressive effect by destroying the Th1/Th2 balance, inhibiting Th17 cell differentiation, and promoting regulatory T (Treg) cell generation. Earlier studies have shown that arsenic exposure affects the number of CD4^+^ T cells and the CD4^+^/CD8^+^ ratios in the spleen and thymus^17^. Arsenic exposure may also lower the Th1 immune response and compromise medication effectiveness to successfully alleviate the intracellular *L. donovani* pathogen^18^. A study in Sudan found that IL-10 and IFN-γ gene polymorphisms were linked to the development of PKDL; immunological polymorphisms contribute to insufficient Th1 immune response to eliminate the *L. donovani* infestation^18,19^. This study comprehensively explored the insight of immunological and pathophysiological consequences of arsenic on the preclinical VL model at the equivalent level of arsenic as reported in the VL endemic area of Bihar, India. This is the unique and first of its kind against visceral leishmaniasis on experimental animal models, as we are reporting through this study.

## Results

### Arsenic exposure exacerbates the *Leishmania donovani* infections

**Figure 1:**
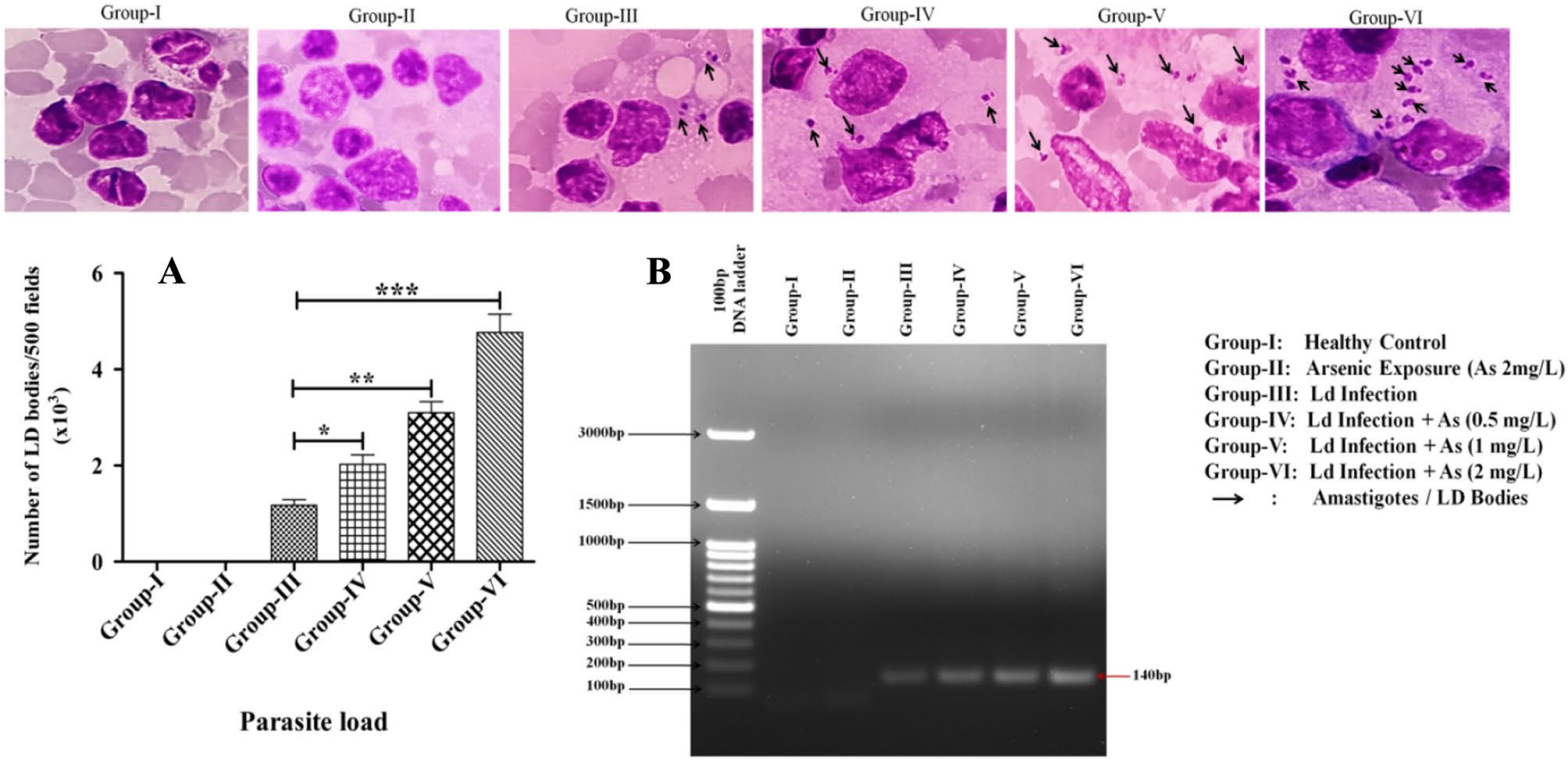
Arsenic exposure upsurges the degree of *Leishmania* infection (Amastigotes/LD bodies; Pics-1). The parasite load was determined in the spleen imprint of experimental VL mice and quantified LD bodies/amastigotes expressed through a bar diagram (Fig. 1A) as well as through PCR (Fig. 1B) by using kDNA specific primer as detailed in Table 3. The data are expressed as mean ± SEM of independent experiment and significance is determined by paired t test.

Arsenic poisoning in groundwater and its effects on human health have been characterized as one of the world's most catastrophic natural groundwater calamities. The experimental role of arsenic exposure on splenic parasitic load was comparatively investigated. The parasitic burden in the spleen of arsenic exposed (2 mg/L) groups of the VL model was analyzed microscopically and showed significantly more (4.15-folds) parasite burden compared to their unexposed counterpart as expressed (Fig. 1). Further, the parasite burden in the spleen of 1 mg/L and 0.5 mg/L arsenic-treated groups was 2.63-fold and 1.7-fold higher than the control group. Additionally, the presence of *Leishmania* parasites was confirmed using PCR with a parasite-specific primer targeting kDNA (Fig. 1B). The increase in parasitic load in arsenic exposed groups indicates that arsenic exposure exacerbates the infection in a dose-dependent manner and may make the host more vulnerable to other opportunistic infectious agents.

### Arsenic exposure alters the host's biochemical characteristics

Subjects/Individuals exposed to elevated levels of arsenic from polluted water negatively impacted the host's various physiological and biochemical characteristics. Hence, the effect of arsenic exposure on potent host biochemical variables in the experimental VL mice model was studied. The observed results showed significantly higher levels of AST (2.3-fold), ALT (3.45-fold), ALP (3.82-fold), BUN (2.06-fold), Creatinine (2.66-fold), and Urea (2.74-fold) in 2 mg/L arsenic exposed groups than that of unexposed healthy control as detailed in Table 1. Thus, these results revealed increased arsenic concentration in drinking water correlated to malfunctioning kidneys and liver in the VL host.

**Table 1:** Exposure to arsenic amplifies hepatonephric toxicities, leading to detrimental effects on the liver and kidneys. In order to evaluate the effect of arsenic on potent biochemical variables, serum samples were collected from all the experimental groups of mice. Following this, we proceeded to quantify essential biochemical markers, and the obtained outcomes from these analyses comprehensively presented in Table 1.

Parameters	Group-I	Group-II	Group-III	Group-IV	Group-V	Group-VI
ALT(iu/l)	85 ± 9	90 ± 15	87 ± 12	94 ± 14	136 ± 9	285 ± 14
AST(iu/l)	17 ± 4.4	33 ± 4.2	25 ± 7.3	32 ± 5.2	34.7 ± 7.6	37.6 ± 3.45
ALP(u/l)	167 ± 27	227 ± 32	192 ± 29	235 ± 24	427 ± 42	627 ± 34
BUN (mg/L)	7.9 ± 2.3	9.1 ± 2.2	7.85 ± 3.4	9.7 ± 2.75	14.2 ± 2.46	16.3 ± 2.2
Urea (mg/L)	12.4 ± 1.9	15.3 ± 3.72	12.7 ± 4.2	16.1 ± 3.32	22.9 ± 4.3	34 ± 6.4
Creatinine(mg/dl)	0.75 ± 0.2	1.02 ± 0.57	0.97 ± 0.34	1.04 ± 0.23	1.12 ± 0.62	2.06 ± .47

### Arsenic exposure reduces hepatic thiol levels

**Figure 2:**
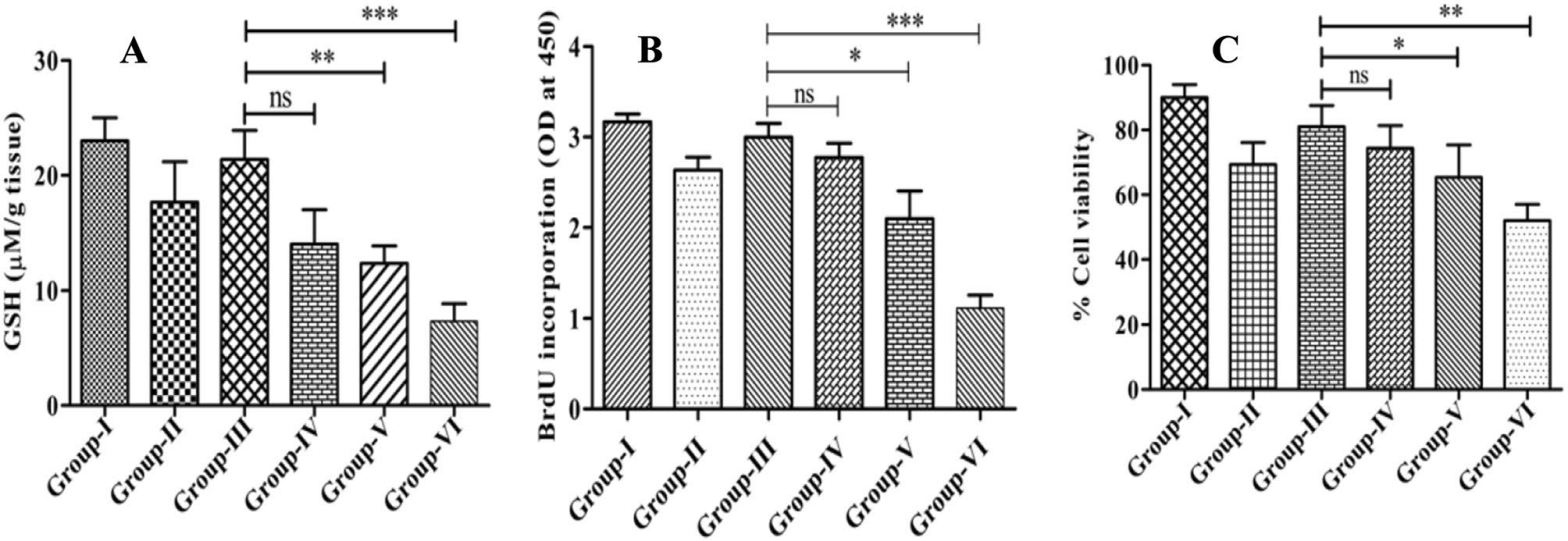
Arsenic exposures dampen the hepatic GSH levels and lymphoproliferative responses. (**A**) Degrees of reduced hepatic thiol in arsenic exposed and unexposed animals are demonstrated in Fig. 2A. (**B**, **C**) The incorporation of BrdU and MTT cell viability in splenocytes isolated from various groups of mice, including those exposed and unexposed to arsenic, at a specific time, and the resulting data is presented through a bar diagram respectively.

Intracellular reduced thiol play a central part in maintaining cellular redox equilibrium. Its ability to combat harmful substances, like arsenic, showcases their critical role in cellular defense mechanisms. This process highlights the remarkable adaptation of cells to counteract threats and maintain their functionality amidst challenges posed by environmental toxins like arsenic. The experiments evaluated reduced hepatic thiol in the arsenic exposed and unexposed mice VL model using the spectrophotometric technique. The observed result showed a substantial level of down-regulation in reduced hepatic thiol in arsenic exposed mice (2.92-fold, 2 mg/L; 1.83-fold, 1 mg/L; 1.5-fold mg/L) than that of the unexposed group (Fig. 2A).

The decreased expression of reduced hepatic thiol could limit the bio-inactivation or detoxification of arsenic or related vicious substances; in turn, the health of individuals could be compromised and at high risk of attack by malicious agents.

### Arsenic exposure limits the Lymphoproliferative responses

Lymphocytes are the vital component of cellular immunity, and monitoring their proliferation enables us to know the insight magnitude of immune responses. Tracking lymphocyte proliferation involves analyzing the increase in their numbers, which reflects the body's attempt to neutralize the threat. This information helps us comprehend how vigorously the body is combating an infection or disease. This knowledge enhances our ability to recognize the body's defense mechanisms, enabling us to design targeted interventions and treatments to bolster immunity and combat diseases effectively. The lymphoproliferative response was comparatively assessed in the arsenic exposed and unexposed VL mice model using BrdU incorporation assay. The observed results showed that arsenic exposure significantly lowered the T cell proliferation by 2.6-fold and 1.42-fold in the 2 mg/L and 1 mg/L arsenic-exposed mice group than in unexposed group. These findings were congruent with the results of MTT studies, whereas the untreated healthy group served as a negative control (Fig. 2B).

### Arsenic exposure modulates Th1/Th2 cytokines produced by T lymphocytes

Cytokine has a central role in inducing T cell phenotype (Th1 and Th2) and effectively generating immunological responses to defend against intracellular pathogens like *Leishmania* parasites and viral infections. Cytokines play a pivotal role in orchestrating immune responses by influencing the differentiation of T cells into distinct phenotypes, namely Th1 and Th2 cells. Th1 cells are prompted by specific cytokines to initiate responses against intracellular pathogens and, fostering cell-mediated immunity and activating macrophages to engulf and eliminate infected cells. Conversely, Th2 cells generate a different response under the guidance of distinct cytokines. They primarily target extracellular threats and are integral in allergic reactions and defense against helminth parasites. Understanding the interplay of cytokines and T cell phenotypes is crucial for tailoring effective immune responses.

**Figure 3:**
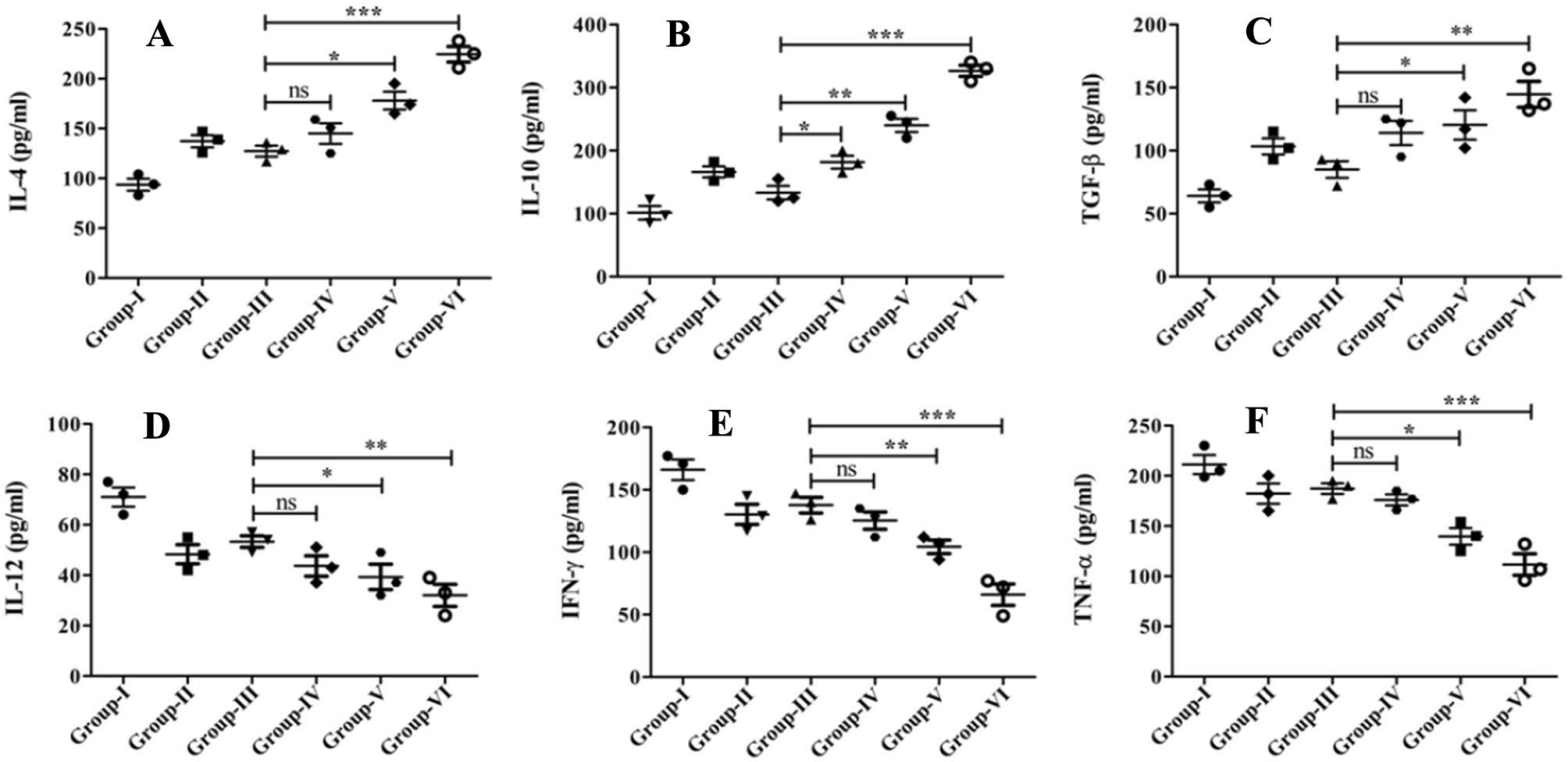
Arsenic exposure pronounced the pro-leishmaniasis Th2-subtype of an immune response. Expression of Th2 cytokines likes (**A)** IL-4; (**B)** IL-10; (**C)** TGF-β was significantly up-regulated in the arsenic-exposed group compared to the unexposed counterpart. Further, the levels of Th1 cytokines such as (**D**) IL-12; (**E**) IFN-γ; (**F)** TNF-α, were also quantified using the ELISA technique in splenocytes isolated from a various group of experimental mice. The obtained results are illustrated using a scatter plot. The experiments were carried out three times each, and statistical significance was assessed using a one-way ANOVA followed by Tukey’s post hoc multiple comparison tests.

We, therefore, comparatively evaluated both Th1 and Th2 cytokines in splenocytes of arsenic exposed and un-exposed BALB/c mice. In continuation to this, an increased amount of Th2 cytokines like IL-4 (2.4-fold), IL-10 (3.25-fold), and TGF-β (2.2-fold) were observed in arsenic exposed (2 mg/L) groups than unexposed. In addition, the amount of Th1 cytokines such as IL-12 (2.2-fold), IFN-γ (2.5-fold), and TNF-α (1.9-fold) was lower compared to the arsenic-unexposed groups (Table 2; Fig. 3). These results show that the immune response triggered in the arsenic exposed mice group was pronounced the pro-leishmaniasis Th2-subtype of an immune response, further deteriorating the host's protective immunity and fuelling up the VL menace.

**Table 2:** This table demonstrates the quantitative assessment of Th1 and Th2 cytokine levels in arsenic-exposed and unexposed animals across different groups. Conversely, there is a subtantial rise in Th2 cytokine levels, indicating an amplification of this specific immune response components among the animals exposed to arsenic. These distinct regulatory patterns of cytokine expression are illustrated in the table provided.

Variables	IL-4	IL-10	TGF-β	IL-12	IFN- γ	TNF-α
Group-I	92 ± 7	101 ± 9	64 ± 7	73 ± 13	166 ± 26	211 ± 32
Group-II	142 ± 9	156 ± 14	102 ± 12	48 ± 6	129 ± 14	182 ± 21
Group-III	127 ± 11	133 ± 16	85 ± 11	53 ± 9	141 ± 11	188 ± 15
Group-IV	145 ± 23	178 ± 24	114 ± 15	44 ± 5	126 ± 23	176 ± 19
Group-V	176 ± 15	245 ± 32	117 ± 21	39 ± 17	105 ± 17	139 ± 26
Group-VI	224 ± 17	327 ± 41	145 ± 41	32 ± 8	67 ± 14	106 ± 13

### Arsenic exposure restrains macrophage effector functions

**Figure 4:**
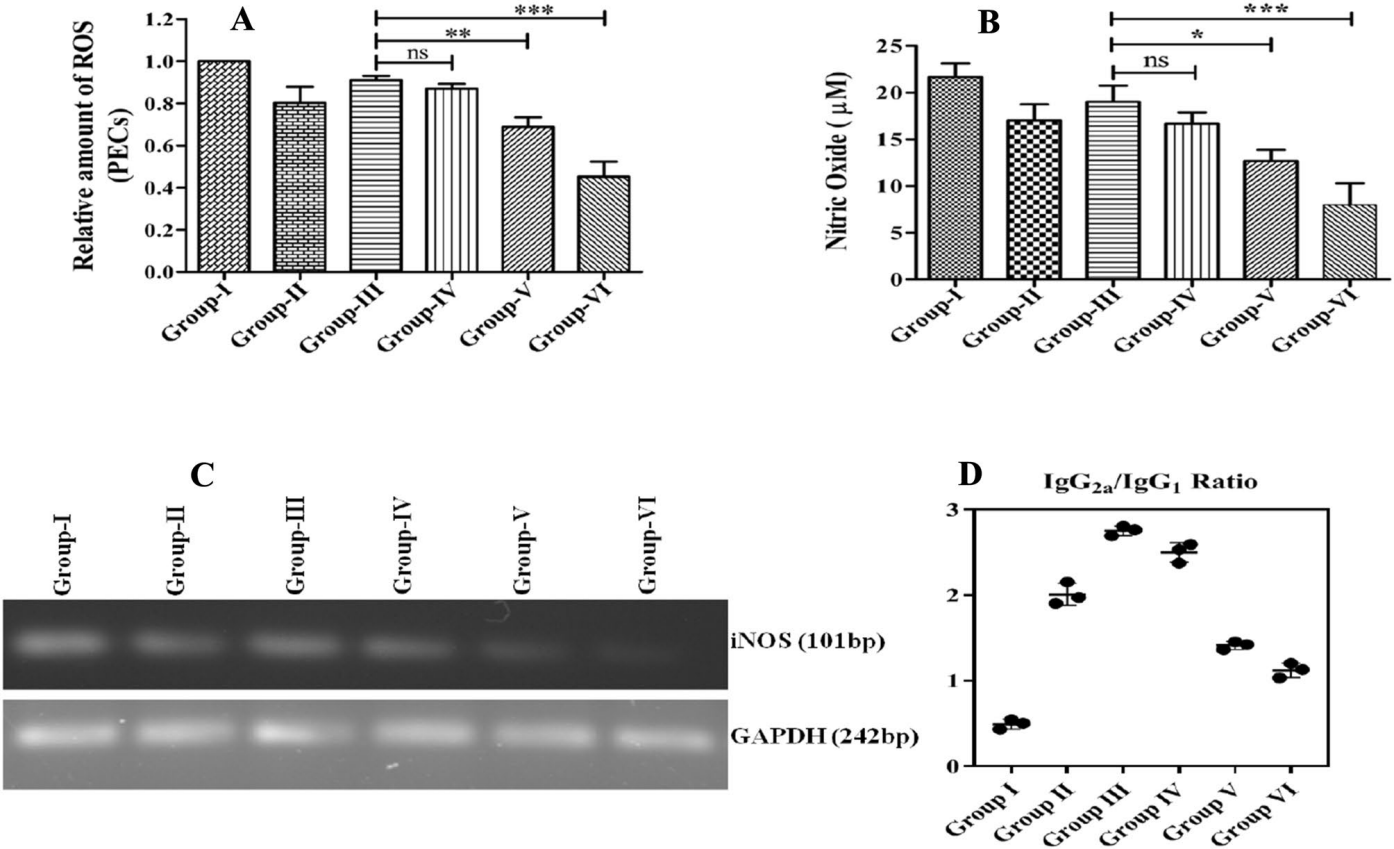
Evaluation of the generation of anti-leishmanial effector molecules viz. reactive oxygen species (ROS), nitric oxide (NO), and inducible nitric oxide synthase (iNOS) from mice belonging to each group of arsenic exposed and unexposed animals. (**A**) ROS production in cultured various mice groups. (**B**) NO release in splenocytes cultured supernatant. (**C**) Qualitative assessment of iNOS and GAPDH as control (the complete image of gel is given in supplementary information Fig. S1). (**D**) To examine the Th1/Th2 cross-regulation and to comprehend the underlying mechanism, we employed indirect ELISA to estimate the serum levels of parasite-specific IgG isotypes. Following the specified experimental period, blood from all experimental mice groups was collected to isolate serum to perform ELISA. The observed IgG2a/IgG1 isotype ratios for Group IV (1.2-fold), Group V (1.45-fold), and Group VI (2.53-fold) were lower compared to mice infected with *Leishmania* parasites (Group III). These results suggest a positive association between arsenic exposure and modulation in host protective immunity in VL subjects, potentially contributing to increased *Leishmania* parasite survival and exacerbation of VL.

Macrophage cells have the inherent ability to express and induce ROS, NO, and iNOS release in response to various stimulation/phagocytosis to kill intracellular pathogens like the *Leishmania* parasite imprisoned in the phagolysosomes. The confinement of *Leishmania* parasites within phagolysosomes provides a conducive environment for macrophages to carry out their defensive actions. The aforementioned noxious molecules function synergistically to incapacitate the fundamental cellular mechanisms of the pathogens, thereby impeding their proliferation and ultimately culminating in their eradication. Then subsequent experiments were performed to measure the intensity of ROS, NO, and iNOS in all the experimental mice groups. The arsenic exposed (2 mg/L) VL mice groups produce significantly less ROS (2.2-fold), NO (2.65-fold), and iNOS than the unexposed control groups (Fig. 4A–C). The down-regulation in oxidative and nitrosative burst reveals that arsenic exposure favors the parasite survival inside the host macrophage cells, resulting in aggressive disease conditions.

### Arsenic exposure induces a lower IgG2 to IgG1 ratio in VL

To investigate the Th1/Th2 cross-regulation and comprehend the humoral immune responses, indirect ELISA was used to identify the presence of *Leishmania*-specific IgG isotypes in the mice serum belonging to different experimental groups. The mice infected with *Leishmania donovani* and exposed to varying concentrations of arsenic (0.5 to 2 mg/L) exhibited higher levels of IgG1 than IgG2a compared to naive healthy groups (group-I), and only *L. donovani* infected mice groups (group-III) (Fig. 4D). The observed IgG2a to IgG1 ratio was 1.2-, 1.45-, and 2.53-fold less in 0.5 mg/L, 1 mg/L, 2 mg/L, respectively, in the arsenic exposed groups compared to the unexposed group (Fig. 4D). These isotypes data suggest a potential correlation between arsenic and modulation in host protective immunity, resulting in parasite survival, development, and exacerbation of VL in individuals exposed to arsenic daily.

### Arsenic exposure modulates MAPK signaling in the VL host

**Figure 5:**
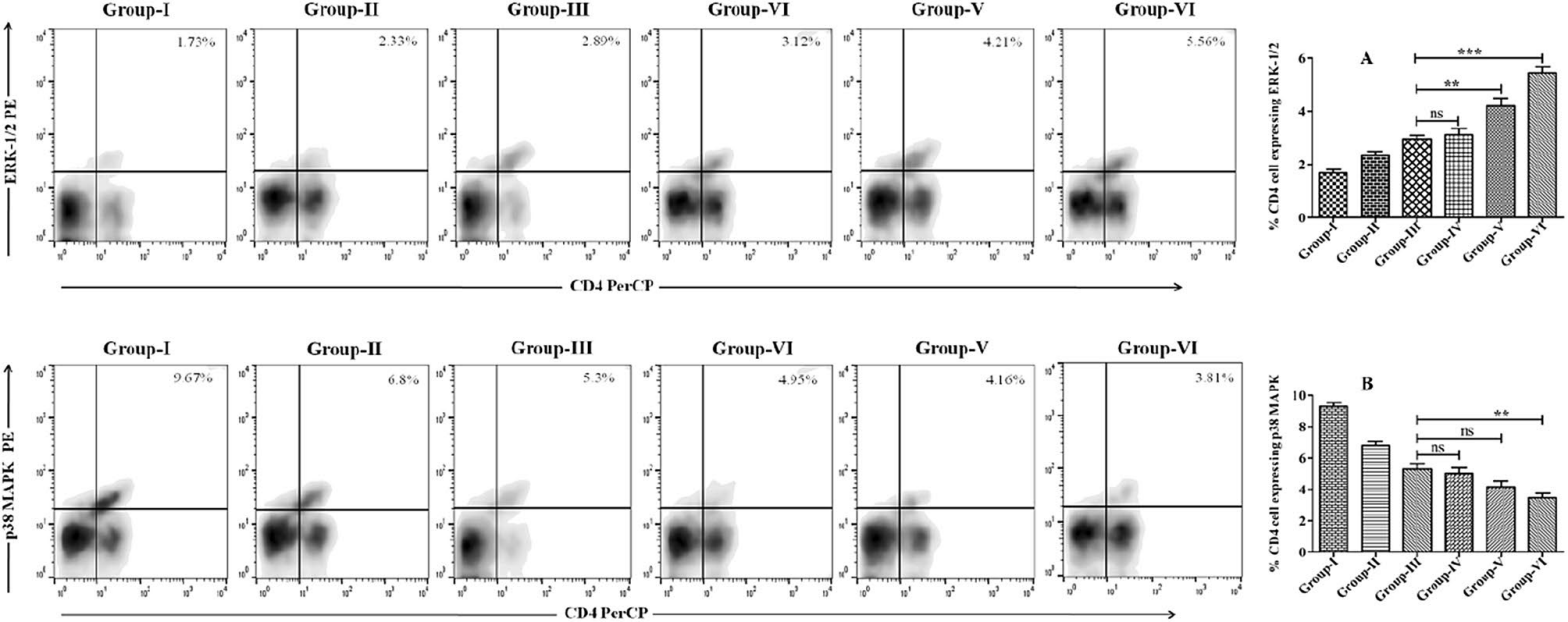
Modulation of MAPK signaling cascade as determined by flow cytometry in CD4 + T cells in arsenic-exposed and unexposed mice groups. (**A**) Percentage CD4 + T-cells co-expressing phosphorylated ERK-1/2 in arsenic exposed and various unexposed groups of mice. (**B**) The proportion of CD4 + T-cells showing phosphorylated p38 MAPK in mice-derived splenocytes of CD4 + T-cells of different groups of mice.

The mitogen-activated protein kinases (MAPK) are involved in the integration and processing of various extracellular signals and their activation elicits multiple biological reactions^20^. The activation of the extracellular regulated kinase 1/2 (ERK-1/2) is linked with pro-*Leishmania* cytokines like IL-10 and TGF-β, whereas p38-MAPK activation transduces signals that kill intracellular pathogens, including the *Leishmania* parasite^21,22^. In this context, the potential impact of arsenic on MAPK family proteins has been comparatively studied by employing flow cytometry (FACS). The activation trends of two MAPK family proteins, viz. ERK-1/2 and p38-MAPK showed a contrasting pattern in arsenic exposed groups compared to unexposed control. The intensity of ERK-1/2 expression was up-regulated by 3.2-fold as compared to control. However, the expression of p38-MAPK was down-regulated by 2.55-fold in the same experimental conditions (Fig. 5). These results suggest that arsenic exposure may induce an increase in ERK-1/2 expression while concurrently suppressing the p38-MAPK signaling cascade, resulting in an aggravation of visceral leishmaniasis (VL) in individuals subjected to continuous arsenic exposure.

## Discussion

In view of the published reports of various surveys, journals, and websites, India and its subcontinent are in the throes of the worst water crisis in its history, as the critical groundwater resource is continuously getting contamination by various means of natural and anthropogenic activities. Groundwater contamination is one of the major sources of human exposure to arsenic, and significantly wreaked havoc the human health and other living entities globally and substantially exacerbated the VL condition, especially in India and its subcontinent^23–25^. Groundwater arsenic pollution with VL and antimonial resistance appears predominantly in the Indian subcontinent. Arsenic has long been recognized to cause cross-resistance to SSG and immunosuppression in vitro^10,13^. However, this phenomenon has still not been demonstrated in vivo in the VL host, particularly at the reported levels of arsenic in groundwater and biological samples of the hyper-endemic area of visceral leishmaniasis in Bihar, India.

This study extensively investigated and showed distorted humoral and cellular protective immunity in arsenic exposed VL mice in a dose-dependent manner: it also induced hepatonephric toxicity with increased splenic *Leishmania donovani* burden. However, the use of the mouse model has long been debated in terms of human relevancy. It differs somewhat but shares many functional similarities in the immune response. As a result, it's a sound model system for studying immunological responses, and it may be appropriately generalized to human concerns^26,27^. Accordingly, the expression and release of leishmanicidal mediators such as ROS, NO, and iNOS were investigated and shown as reduced in arsenic-exposed experimental groups (Fig. 4A–C). Macrophages largely generate these messengers to alleviate the intracellular amastigotes and are critical to the controlled or exacerbated response that culminates in clinical manifestations^28^. Meanwhile, the *Leishmania* parasite count/LD bodies in the spleen of BALB/c mice were microscopically quantified and found higher in arsenic-exposed groups than in the unexposed VL control group (Fig. 1). The suppressed expression of oxidative and nitrosative bursts logically supports higher parasite load, infectivity, and pathogenesis, resulting in disease exacerbations. The increase in splenic parasite load corresponded to a recent study that found a rise in viral (50-fold) and bacterial (17-fold) load in zebra fish exposed to 2 and 10 ppb arsenic; both the doses considered safe levels in drinking water. The increased pathogen loads were due to the damped fish's overall innate immune health, as shown by lower respiratory burst activity^29^. In another study, authors have demonstrated that an ecologically appropriate amount of arsenic exposure increased the differentiation of regulatory T (T_reg_) cells by lowering the cytokine secretion from splenocytes to induce immunosuppression in experimental mice, which in turn increased susceptibility to *Mycobacterium fortuitum* infection^26^. In addition, we observed significant levels of splenomegaly and hepatomegaly in the arsenic-exposed VL mice, prompting us to investigate hepatic and renal function extensively. The detected serum levels of AST (2.3-fold), ALT (3.45-fold), ALP (3.82-fold), BUN (2.06-fold), Creatinine (2.66-fold), and Urea (2.74-fold) were significantly higher in 2 mg/L arsenic-exposed VL mice groups than those of arsenic unexposed group, as shown in Table 1. This observation advocated for the etiology of arsenic poisoning or arsenicosis and related clinical manifestation of liver and kidney damage in the VL host. The liver is the principal organ responsible for arsenic metabolism^30^. Previously, it was reported that most of the consumed arsenic cleared from the body within an hour: but some parts are processed through the liver and express its mutilating effects on thiol metabolism^31,32^. The observed hepatic reduced thiol was 2.92-fold less in arsenic exposed (2 mg/L) experimental VL than that of the unexposed group (Fig. 2A); possibly, this could intensify the *Leishmania donovani* infection, as evident from our data (Fig. 1). Based on these observations, another explanation for VL aggravation in arsenic exposed settings is that the *Leishmania* parasite may shatter the major trypanothione synthase (TSH2) pathway and develop evasive mechanisms, resulting in the promotion of infections by negatively affecting the abrupt release of leishmanicidal molecules^33,34^. Suppressed oxidative stress could be a possible outcome of up-regulated *Leishmania’s* reduced thiol that acted as a defense system to counteract the xenobiotic molecules generated against the *Leishmania* parasite^13,35^.

Due to all these changes in arsenic-exposed VL hosts, it would be exciting and worthwhile to explore the immunomodulatory consequences of arsenic exposure to VL host and its associated repercussions. The study began by evaluating lymphoproliferative responses upon arsenic exposure, principle armor against *Leishmania* parasites infections, especially pronounced Th1 responses. T cell proliferation and differentiation were assessed and found 2.6-fold less in the arsenic exposed group (2 mg/L) than in unexposed groups (Fig. 2B). T cell, a prerequisite for cell-mediated immunity and pivotal in macrophage (Mφ) activations; activated Mφ elicited the anti-leishmanial activities to eliminate intracellular pathogens, including *Leishmania* amastigotes. Cytokines like IL-12, IFN-γ, and TNF-α were predominantly expressed from macrophages cells and well-coordinated in macrophage activation to induce leishmanicidal factors, such as ROS, NO, and iNOS^36–38^. However, the quantitative analysis of these cytokines appeared to be checked by IL-12 (2.2-fold), IFN-γ (2.5-fold), and TNF-α (1.9-fold) in arsenic-exposed groups (Fig. 3D–F). Whereas the degrees of Th2 cytokines like IL-4 (2.4-fold), IL-10 (3.25-fold), and TGF-β (2.2-fold) were up-regulated as observed in arsenic exposed (2 mg/L) groups compared to an unexposed group (Fig. 3A–C; Table 2). Concomitantly, the humoral immune response was examined by evaluating the expression of *Leishmania-*specific IgG1 and IgG2 antibodies in sera of the arsenic exposed and un-exposed VL host. IgG1 isotypes expression is related to overall Th2 cytokines (IL-4, IL-10) response, resulting in disease aggravation, whereas IgG2a antibodies were generated in the presence of disease resolving pro-inflammatory cytokines like IL-12, IFN-γ, and TNF-α^39,40^. The preponderance of IgG1 isotypes over IgG2a in arsenic exposed VL host advocated for the disease progression via immunopathogenesis (Fig. 4D). Moreover, the activation and suppression of various signal transduction-related kinases, such as p38 MAPK and ERK-1/2, are crucial to controlling or aggravating the *Leishmania* response that manifests in clinical presentation. In continuation to this, we have observed the significant up-regulation in the expression of ERK-1/2 and down-regulation of p38 MAPK in arsenic exposed VL host compared to the unexposed group (Fig. 5). Previously some author demonstrated that *L donovani* lipophosphoglycan induced ERK to inhibit the production of IL-12 and NO, significantly promoting survival; thus, inhibition of ERK is expected to decrease parasite survivability^41,42^. The activation/phosphorylation of ERK-1/2 promotes the generation of IL-10, which in turn supports intracellular *Leishmania* parasite proliferation and disease advancement. Alternatively, activation of p38-MAPK is attributed to increased production of iNOS, ROS, and IL-12; these factors are crucial for alleviation of intracellular *Leishmania* amastigotes. According to these data and previous studies, the decreased expression and phosphorylation of p38-MAPK have reciprocally associated with phosphorylation of ERK-1/2 in *Leishmania* infected cells, resulting in the promotion of *Leishmania* parasites viability, immune evasion, and sabotage of the host.

This study demonstrated that arsenic exposure at the equivalent of the reported concentration of arsenic in the VL hyper-endemic area of Bihar, India, negatively affected the host's protective response, allowing *Leishmania donovani* to orchestrate inside the host. As the *Leishmania donovani* evolved some evasive mechanism through suppression of host reactive intermediate species and Th1 responses to mount the hepatonephric toxicity, over expression of *Leishmania* reduced thiol had also been contributed to disease progression and immunosuppression. The present study suggests that the contorted Th1-subtype and exacerbated Th2-subtype immune responses are involved in the enhanced susceptibility and pathogenesis of *Leishmania* parasite among individuals regularly exposed to environmentally relevant arsenic level. Moreover, the study's findings could contribute to resolving antimony (Sb) resistance linked to arsenic exposure in VL endemic areas. One possible approach is to combine SSG with potential host-targeted therapeutics in a chemo-immunotherapeutic strategy aimed at strengthening and enhancing Th1 responses to improve anti-leishmania effects.

## Materials and methods

### Animal ethical clearance

All procedures were conducted in compliance with the ARRIVE guidelines. The Institutional Animal Ethics Committee (IAEC) of ICMR-Rajendra Memorial Research Institute of Medicals Sciences (letter No: RMRI/ICMR/AH/416/2017-18) Patna, India had, approved this study before starting the work on experimental animals. The inbred BALB/c mice were purchased from CSIR-CDRI, Lucknow, India, and maintained in the animal house of ICMR-RMRIMS, Patna, under hygienic conditions and provided with balance pellet diet and water *ad-libitum*.

### Oral arsenic exposure to BALB/c mice

The different concentrations of arsenic to be administered to experimental mice were chosen to an equivalent to the levels of arsenic concentration reported in VL hyper endemic area of Bihar, India^43–45^. For this, four to six-week-old, inbred male BALB/c mice were procured, divided into six different groups consisting of 10 mice in each group (Group I-VI).

- Group-1: Healthy Control (Naive control).
- Group-II: Arsenic exposure (As 2 mg/L).
- Group-III: *L donovani* infection without arsenic exposure (Infection control).
- Group-IV: 0.5 mg/L arsenic exposure (As 0.5 mg/L) followed by *L donovani* infection.
- Group-V: 1 mg/L arsenic exposure (As 1 mg/L) followed by *L donovani* infection.
- Group-VI: 2 mg/L arsenic exposure (As 2 mg/L) followed by *L donovani* infection.

These groups were exposed to specified arsenic concentrations through their drinking water for one month (30 days) before inoculating *Leishmania donovani* parasites; and continued until day 90 (day of animal sacrifice), except for Group-1 and Group-III, which served as a negative control. Arsenic was dissolved in Milli-Q water as a stock, followed by dilution in drinking water *ad-libitum* at a concentration range between 0.5 mg/L to 2 mg/L; drinking water with or without arsenic changes daily.

### *Leishmania donovani* culture and infection establishment in BALB/c mice

The reference strain of *Leishmania donovani* promastigotes (MHOM/IN/1983/AG83) AG83 was procured from the cryobank repository of the ICMR-RMRIMS, Patna, India. The procured *Leishmania* parasites were grown and maintained in RPMI-1640 medium (Sigma-Aldrich) added with antibiotics (Penicillin, Streptomycin, and Gentamicin) and 10% heat-inactivated fetal bovine serum (FBS), followed by incubation at 24 ± 1 °C to facilitate biological oxygen demand (BOD). Next, highly motile and infective metacyclic *L. donovani* promastigotes were prepared to infect male BALB/c mice intraperitoneally with an inoculum size 2 × 10^7^ cells/ml; 200 µl/animal. After eight weeks of injection, on day 60, mice were euthanized by CO_2_ asphyxiation to harvest the spleen. The harvested spleen was imprinted on glass slides, followed by methanol fixation and Giemsa staining for microscopic examination to quantify the intracellular splenic amastigotes burden under the 100 × oil immersion field as detailed earlier^46^. Furthermore, the presence of *Leishmania* parasites was validated by PCR using kDNA-specific primers, as detailed in Table 3. Also, a part of the spleen was crushed and suspended in culture media (NNN and RPMI-1640), followed by incubation in a BOD at 24 ± 1 °C for conversion of amastigotes into promastigotes to confirm the infection.

**Table 3:** In this study, we analyzed the expression of the iNOS gene in mice and the kDNA of *Leishmania* parasites. This analysis included specific primer sequences, along with corresponding annealing temperatures and the size of the resulting amplification product (amplicon size). GAPDH was used as a reference in these investigations.

Gene	Primer sequence	Annealing temp (°C)	Amplicon size (bp)
iNOS	F_5-CCCTTCCGAAGTTTCTGGCAGCAGC-3 R_5-GGCTGTCAGAGCCTCGTGGCTTTGG-3	56	101
GAPDH	F_5-CAAGGCTGTGGGCAAGGTCA-3 R_5-AGGTGGAAGAGTGGGAGTTGCTG-3	56	242
*Leishmania* kDNA	F_5-CTTTTCTGGTCCTCCGGGTAGG-3 R_5-CCACCCGGCCCTATTTTACACCAA-3	56	140

### Biochemical assessment

The retro-orbital/tail vein blood from each experimental group of BALB/c mice was collected into plain tubes (without any anti-coagulant) for biochemical study for serum preparation, as described elsewhere with some modifications^47,48^. The collected blood specimens were subjected to incubation at 37 °C for an hour to settle down the cells, and subsequently, samples were centrifuged at 700 g for 15 min to harvest the serum. The collected serum was subjected to ALP, ALT, AST, BUN, Creatinine, Urea, etc., investigations using Semi-Auto Biochemistry Analyser (Merck Microlab-300) with reagents supplied by Coral Clinical Systems (Tulip Diagnostic, Pvt. Ltd.) India.

### Hepatic reduced thiol estimation

The total reduced thiol was determined in deproteinized lysates of hepatocytes from each group of the experimental model of BALB/c mice; following Ellman procedures with slight modification as detailed previously^49,50^. In brief, the mice from each group were sacrificed, and the liver was harvested, followed by homogenization and centrifugation to prepare the single-cell suspension of liver cells (hepatocytes). Next, the prepared hepatocytes were suspended in 750 µl of 5% trichloroacetic acid and freeze-thawed for two cycles; denatured proteins and cell debris were removed by centrifugation at 12,000*g* for 10 min. The thiol content in the supernatant was measured by adding DTNB solution (Ellman’s reagent) followed by absorbance recording on a spectrophotometer at 412 nm.

### T lymphocytes proliferation assay

The lymphoproliferative responses in the arsenic-exposed and unexposed group were comparatively investigated against murine-derived spleen cells by employing BrdU and MTT assays as we described earlier with slight changes^51^. In brief, spleens from experimental BALB/c mice were harvested and processed to extract the single-cell suspensions of spleen cells and suspended in the RPMI-1640 medium supplemented with 10% FBS (complete media). Cells number was adjusted (1 × 10^6^ cells/ml) and cultured in a cell culture plate (96-well) and placed in a CO_2_ incubator for 72 h at 37 °C in a CO_2_ incubator. After-incubation, the BrdU incorporation test was employed to monitor the lymphocyte or T-cell proliferation using the BrdU cell proliferation ELISA kit (ab126556) according to the manufacturer's recommendations. Simultaneously, the MTT assay was used in another set of an experiment to monitor the cell viability, for this MTT reagent (5 mg/ml) was added to each well of the cell culture plate; next, MTT solubilizing reagents were added to solubilize the formazan crystal, and then absorbance was recorded at 570 nm on a spectrophotometer (Bio-Rad).

### Extracellular cytokines estimation

The experimental mice from each group were subjected to peritoneal exudates cells (PECs) and splenocytes isolation, as we defined previously, with some modifications^13^. Briefly, the freshly prepared PECs and splenocytes (1 × 10^6^ cells/ml) were plated in two separate 24-well culture plates and kept in a CO_2_ incubator at 37 °C (5% CO_2_ and 95% humidity) for 48 h. Post 48 h, cell-free supernatant was collected by centrifugation at 700*g* for 15 min at room temperature (24 ± 1 °C). Next, the level of various cytokines viz. IL-4, IL-10, IL-12, IFN-γ, TNF-α, and TGF-β were estimated using commercially available ELISA kits (BD OptEIA™, ELISA Kit) as per the manufacturer's instruction. The detected levels of cytokines were expressed in picograms (pg/ml), based on a generated standard curve as per the manufacturer recommendations using recombinant cytokines as provided in the kits. Each experiment was performed with three biological replicates in triplicates.

### Assessment of NO and iNOS

The PECs and Splenocytes of BALB/c mice were harvested and processed to get single-cell suspensions. Cell number was adjusted (1 × 10^6^ cells/ml) and cultured in 12-well cell culture plates at 37 °C in a CO_2_ incubator with 5% CO_2_ and 95% humidity for the next 48 h. Following the stipulated incubation, the nitric oxide (NO) level in the supernatant of cell culture was estimated using a Griess reagent-based nitric oxide assay kit (Thermo-Fisher™) as per the manufacture protocol. Absorbance was measured at 540 nm with a spectrophotometer, and the amount of NO was deduced from the plotted standard curve by using a known amount of sodium nitrite. Coherently, the level of inducible nitric oxide synthase (iNOS) expression was determined by semi-quantitative PCR using primers as detailed earlier^50,52^ (Table 3).

The amplified PCR product was run on 1.5% Agarose gel, stained with ethidium bromide, and eventually quantified and documented on a Bio-Rad gel documentation system.

### Reactive Oxygen species estimation

The levels of intracellular reactive oxygen species (ROS) were monitored, in each groups of experimental mice using 2, 7-dichlorodihydrofluorescein diacetate (H2DCFDA) (Sigma-Aldrich), as previously specified with minor modifications^12^. In brief, the PECs and splenocytes were harvested from euthanized experimental mice and plated in a cell culture plates. Subsequently, the cultured cells were treated with 0.4 mM, H_2_DCFDA (final concentration) for 15 min in the dark. Post incubation, cells were washed twice with PBS and subjected to lyse with lysis buffer (1% Triton X-100 + 1% SDS in 10 mMTris) and monitored the fluorescence intensity at 530 nm (with excitation and emission measurement at 488 and 530 nm, respectively) using a spectrofluorometer. Each test included three repetitions, and the results represent the means and standard deviations of three observations.

### Immunoglobulin evaluation

Upon completion of the stipulated period of experimental duration, blood specimens were collected in a plain vacutainer by cardiac puncture from the arsenic-exposed and unexposed group of mice. Subsequently, the harvested blood was processed to harvest serum to determine the IgG1 and IgG2a immunoglobulin levels by performing an indirect enzyme-linked immunosorbent assay (ELISA), as detailed previously, with some modifications^53^. Briefly, 96-well microtiter plates were coated with 5 μg/mL soluble *Leishmania* antigen (SLA) using bicarbonate buffer (pH 9.5, 100 μL/well) and incubated overnight (12 h) at a temperature of 4 °C. The microtiter plates were then blocked for 1 h with a blocking buffer containing 1X PBS and 5% skim milk to prevent false positive results. Afterward, the microtiter plates were washed three times with a 0.1% Tween-20 in PBS (wash solution), followed by adding primary antibody (mice sera) at a 1:1000 dilution in blocking buffer and incubated for an hour at 37 °C. Next, the microtiter plates were washed five times with washing solution. Subsequently, the HRP-conjugated anti-mouse IgG1 and IgG2a secondary antibodies were applied at a 1:5000 dilution. Finally, 3,3′,5,5′-Tetramethylbenzidine (TMB) was added, and the reaction was stopped with 1N H_2_SO_4_ (50 µL/well), followed by measuring the absorbance at 450 nm using an ELISA reader (Bio-Rad, CA, USA).

### Qualitative assessment of ERK-1/2 and p-38 MAPK proteins

Freshly isolated and prepared splenocytes (1 × 10^6^ cells/well) from the various experimental mice belonging to different experimental groups were cultured and incubated in a CO_2_ incubator at 37 °C with 5% CO_2_ for 24 h. The culture was treated with Golgi-Stop containing brefeldin A (1 µg/ml) (BD Biosciences, USA) for four hours before harvesting. For the fluorescence staining of the surface markers, the cells were incubated with anti-CD4 PerCP specific fluorochrome-tagged antibodies and placed at 4 °C for 30 min. Following the specified incubation period, each cell sample was thoroughly washed with autoclaved PBS by centrifugation at 500*g* for 10 min at 4 °C. Next, the fluorescence-stained cells were treated with Cytofix/Cytoperm solution for 30 min at 4 °C before being treated with 1 ml PermWash buffer (1x; BD Bioscience) and incubation for 5-min at 4 °C. Next, the cells were washed and labeled with intracellular anti-ERK1/2-PE and anti-p38 MAPK-PE antibodies and then incubated for 30 min at 4 °C. Subsequently, 1 × stain solution (PBS with 0.09% NaN3 and 1% FBS) was added to each tube of cell samples and mix-well by gentle pipetting and washed thoroughly by centrifugation at 300*g* for 10 min. Finally, the cell pellet was re-suspended in 500 µl stain buffer and acquired on a FACS Calibur™ (Becton Dickinson, San Diego, USA) equipped with Cell Quest Pro™ software.

### Statistical analyses

The data are expressed as mean ± standard error of the mean (SEM), and statistical analysis was performed using Graph pad prism-5 software. The significance was determined by the Student’s t-test and one-way analysis of variance (ANOVA) with *Tukey’s *post hoc multiple comparison tests. All experiments were run in triplicate, and *P*-value ≤ 0.05 was significant.

### Ethical approval

All procedures were conducted in compliance with the ARRIVE guidelines. This study was carried out following the recommendations of the Animal Ethical Committee of RMRIMS, Patna, India (Letter No.: RMRI/ICMR/AH/416/2017-18). All applicable international, national, and/or institutional guidelines for the care and use of animals were followed.

### Supplementary Information

Supplementary Figures.

## Data Availability

All data generated or analysed during this study are included in this published article and its supplementary information file.
