# Supplementary material for: Arsenic exposure to mouse visceral leishmaniasis model through their drinking water linked to the disease exacerbation via modulation in host protective immunity: a preclinical study
Source: Sci Rep. 2023 Dec 5;13:21461. doi: 10.1038/s41598-023-48642-z (PMC10698031; doi:10.1038/s41598-023-48642-z)
Supplement: Supplementary file 1 — Supplementary Figures. [file 41598_2023_48642_MOESM1_ESM.docx]

**Supplementary Material**


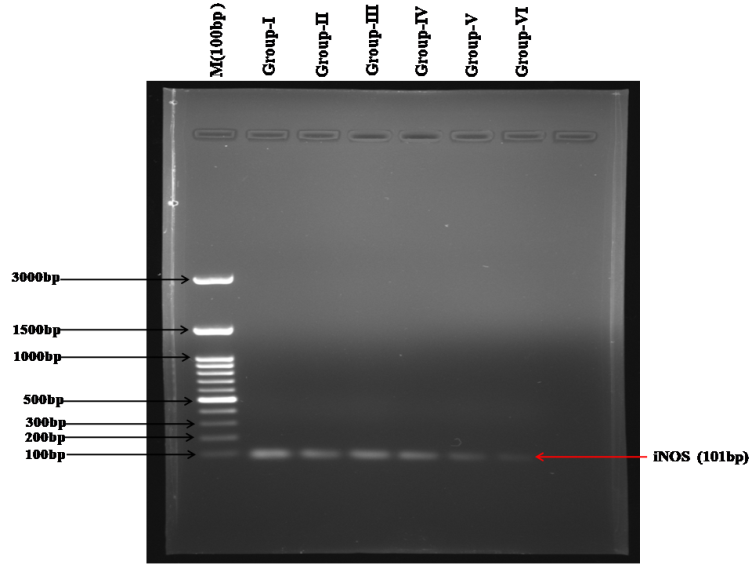

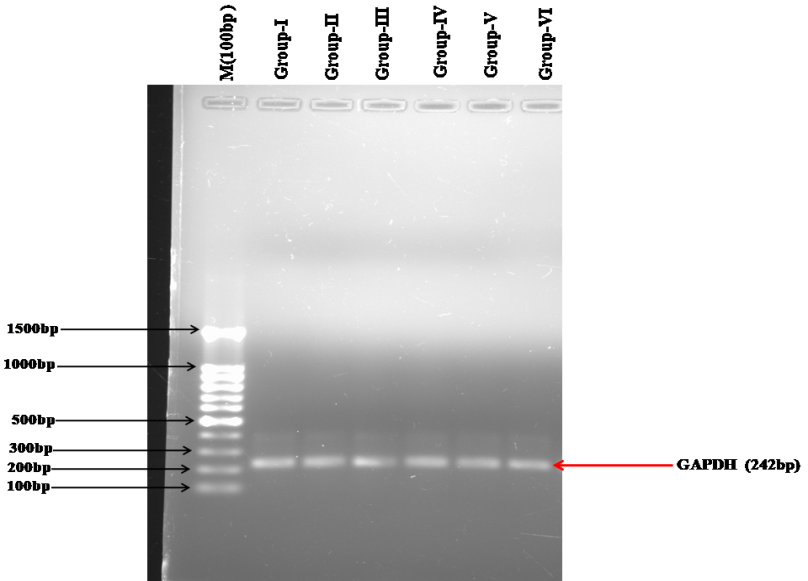


**Figure-S2**

**Figure-S1**

**Figure-S1, S2**: **A qualitative assessment of iNOS (S1), with GAPDH (S2) as reference control.**The expression level of iNOS was measured using semi-quantitative PCR with the gene-specific primers. The amplicon was run on 1.5% agarose gel, stained with ethidium bromide, and documented on a Bio-Rad gel documentation system.


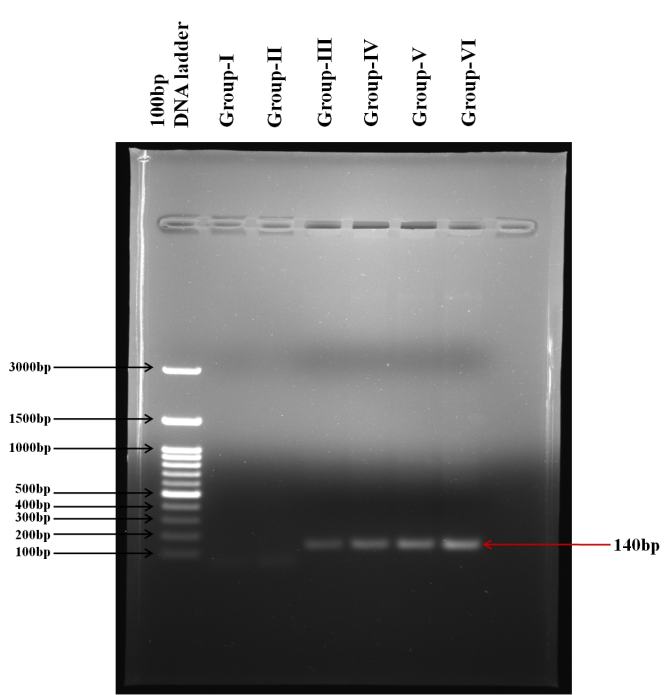


**Figure-S3**

**Figure-S3: A qualitative assessment of *Leishmania* parasites in the spleen of infected mice**. The semi-quantitative PCR was conducted to confirm the presence of *Leishmania* parasites in the spleen of different infected mice group, as detailed in ‘materials and methods’ section of this manuscript, using *Leishmania* kDNA-specific primers, as specified in Table 3.

**Abbreviations:**

**M (100bp):** 100bp DNA ladder

**Group-1**: Healthy Control (Naive control)

**Group-II:** Arsenic exposure (As 2mg/L)

**Group-III:** *L donovani* infection without arsenic exposure (Infection control)

Group-IV: 0.5 mg/L arsenic exposure (As 0.5 mg/L) followed by *L donovani* infection

Group-V: 1 mg/L arsenic exposure (As 1 mg/L) followed by *L donovani* infection

Group-VI: 2 mg/L arsenic exposure (As 2 mg/L) followed by *L donovani* infection.
